# Cholesterol-Based Nanovesicles Enhance the In Vitro Cytotoxicity, Ex Vivo Intestinal Absorption, and In Vivo Bioavailability of Flutamide

**DOI:** 10.3390/pharmaceutics13111741

**Published:** 2021-10-20

**Authors:** Mohamed A. Ali, Magdy I. Mohamed, Mohamed A. Megahed, Tamer M. Abdelghany, Khalid M. El-Say

**Affiliations:** 1 Department of Pharmaceutics and Pharmaceutical Technology, Egyptian Russian University, Cairo 11829, Egypt; mohamed-ali@eru.edu.eg (M.A.A.); mohamed_adel@eru.edu.eg (M.A.M.); 2 Department of Pharmaceutics and Industrial Pharmacy, Cairo University, Cairo 11562, Egypt; magdy.mohamed@pharma.cu.edu.eg; 3 Department of Pharmacology and Toxicology, Al-Azhar University, Cairo 11651, Egypt; tamer.abdelghany@azhar.edu.eg; 4 Department of Pharmacology and Toxicology, Heliopolis University for Sustainable Development, Cairo 11785, Egypt; 5 Department of Pharmaceutics, Faculty of Pharmacy, King Abdulaziz University, Jeddah 21589, Saudi Arabia

**Keywords:** Draper–Lin small composite design, ex vivo intestinal permeation, flutamide, in vitro cytotoxicity, optimization, in vivo pharmacokinetics, prostate cancer

## Abstract

Critical adverse effects and frequent administration, three times per day, limit the use of flutamide (FLT) as a chemotherapeutic agent in the treatment of prostate cancer. Therefore, our research aimed to develop new cholesterol-based nanovesicles for delivering FLT to malignant cells in an endeavor to maximize its therapeutic efficacy and minimize undesired adverse effects. Draper–Lin small composite design was used to optimize the critical quality attributes of FLT-loaded niosomes and ensure the desired product quality. The influence of the selected four independent variables on mean particle size (Y_1_), zeta potential (Y_2_), drug entrapment efficiency (Y_3_), and the cumulative drug release after 24 h (Y_4_) was examined. The optimized nanovesicles were assessed for their in vitro cytotoxicity, ex-vivo absorption via freshly excised rabbit intestine as well as in vivo pharmacokinetics on male rats. TEM confirmed nanovescicles’ spherical shape with bilayer structure. Values of dependent variables were 748.6 nm, −48.60 mV, 72.8% and 72.2% for Y_1_, Y_2_, Y_3_ and Y_4_, respectively. The optimized FLT-loaded niosomes exerted high cytotoxic efficacy against human prostate cancer cell line (PC-3) with an IC_50_ value of 0.64 ± 0.04 µg/mL whilst, it was 1.88 ± 0.16 µg/mL for free FLT. Moreover, the IC_50_ values on breast cancer cell line (MCF-7) were 0.27 ± 0.07 µg/mL and 4.07 ± 0.74 µg/mL for FLT-loaded niosomes and free FLT, respectively. The permeation of the optimized FLT-loaded niosomes through the rabbit intestine showed an enhancement ratio of about 1.5 times that of the free FLT suspension. In vivo pharmacokinetic study displayed an improvement in oral bioavailability of the optimized niosomal formulation with AUC and C_max_ values of 741.583 ± 33.557 μg/mL × min and 6.950 ± 0.45 μg/mL compared to 364.536 ± 45.215 μg/mL × min and 2.650 ± 0.55 μg/mL for the oral FLT suspension. With these promising findings, we conclude that encapsulation of FLT in cholesterol-loaded nanovesicles enhanced its anticancer activity and oral bioavailability which endorse its use in the management of prostate cancer.

## 1. Introduction

Prostate cancer (PCa) is considered the second most widespread carcinoma and the fifth reason for cancer-related death among men around the world [1,2]. Over one-half of males 50 years or older show either histological and/or clinical signs of benign prostatic hypertrophy (BPH) [3]. PCa identification rates show great variation worldwide, with a higher incidence in the USA and Europe than in South and East Asia [4]. In the USA, the frequency rate is estimated to be 119.9 patients per 100,000, whereas in China it is estimated to be 1.6 patients per 100,000 [5]. About 20% of 191,000 PCa cases identified in 2020 in the USA alone were found to be metastatic [4,6]. Uncontrolled proliferation of oncogenic cells in the prostatic tissue under the influence of testosterone and 5α-dihydrotestosterone (DHT) on androgenic receptors is the main characteristic of both PCa and BPH [3,7]. The main challenge in cancer treatment is the high toxicity profiles of most anticancer drugs [8]. Therefore, delivering anticancer drugs to the desired organ in a suitable therapeutic concentration with the highest efficacy and minimal adverse effects remains the highest priority in cancer research [9]. Androgen receptor antagonists such as flutamide (FLT) are widely used in the treatment of hormone-sensitive neoplasms as prostate cancer [10].

FLT is currently used either as a single agent or in combination with Luteinizing hormone-releasing hormone (LHRH) for treatment of both BPH and PCa, also has a significant role in whole androgen suppression therapy and pre-operative as an adjuvant therapy in the entire removal of the prostate gland [11,12]. Being a non-steroidal anti-androgenic agent acts by specifically blocking androgen receptors in the prostatic tissues competitively [12] and accordingly it has the advantage that it is devoid of cardiovascular adverse effects often occur with hormonal therapy with estrogen or steroidal anti-androgen [13,14]. Even with the extensive use of FLT oral tablets in a dose of 250 mg three times daily [15], FLT is categorized according to Biopharmaceutics Classification System (BCS) as a class II drug that possesses poor solubility in water [16]. Furthermore, it is extensively metabolized through first-pass hepatic metabolism which leads to low drug concentration at target sites, lower bioavailability and short half-life (t_1/2_) of about 5–6 h [7,11]. In order to compensate for the short t_1/2_ and lower bioavailability, FLT oral tablets are administrated frequently in a total dose of 750 mg per day which has greater side effects such as reduction of sexual desire and reduction of number and activity of sperms in men, in addition to mild to severe hepatotoxicity [10,17]. Consequently, developing novel drug carrier systems of FLT that aim to (1) improve solubility and permeability thus increase drug concentration at absorption sites which leads to improving bioavailability, (2) improve drug targeting to the desired site without harming other organs and (3) evade first-pass hepatic metabolism, are of great importance [7,11,14].

Nanoscience has proven an excellent achievement in developing novel diagnostic aids and therapies for a variety of diseases particularly cancer [18,19]. Nanocarriers (NCs) usage to target chemotherapeutics toward malignant cells plays a critical role in cancer management [20]. Chemotherapies can be directed toward cancer cells via attachment of NCs to special ligands which capable of binding to particular antigens or receptors on the surface of malignant cells [21,22,23]. Design of nanovesicles with optimal surface charge, particle size and release characteristics improve drugs’ bio-distribution and extend their plasma t_1/2_. Similarly, they can change drugs’ pharmacokinetics without disturbing their therapeutic effects and transport drugs to minute unreachable parts inside the body. Superior penetrability and holding is the foremost benefit of nanocarriers for bringing chemotherapeutics, with effective concentrations to malignant cells than unaffected cells [24]. Niosomes are considered among the promising nanocarriers based on nonionic surface energetic agents which can be used successfully to achieve the previously mentioned purposes [25,26]. Niosomes are the thermodynamically stable double-layered arrangement of nonionic surfactant obtained upon hydration of dried thin film of surfactant in the presence of cholesterol [27,28]. Unlike liposomes that show inadequate entrapment of lipid-soluble drugs, the distinctive geometry of niosomes makes them able to encapsulate both water-soluble and water-insoluble drugs in the hydrophilic core and the bilayer structure, respectively [29,30,31]. Among the advantages of niosomes are: (1) they allow drug delivery in a sustained and/or controlled fashion, (2) they are stable over extended storage periods, (3) highly tolerable showing minimum toxicity and high biocompatibility, (4) they enhance the bioavailability of orally administrated drugs with little bioavailability, (5) they can incorporate drugs that decomposed by gastric acidity or enzymes and protecting them, (6) they can improve t_1/2_ and metabolism of chemotherapies and so, enhance their accumulation in tumors [31,32,33]. Formulation of niosomes via try and error procedure costs effort and money as it depends on the alteration of one factor at a time while maintaining other factors unchanged; optimized techniques are largely replacing old-fashioned methods used in niosomal formulation.

Experimental designs are considered an effective and powerful tool in the formulation of niosomes and other drug transport systems, they allow examining a huge number of factors concurrently in few experimental runs [34,35]. Among the experimental designs used for optimization is the Draper–Lin small composite design (D-LSCD), which is an extremely efficient optimization method that is based on four-factor three-level design and is used to develop mathematical models for the estimation of associations between the dependent and independent variables [36].

The goal of this study is to formulate an enhanced stable FLT-loaded niosomes with optimal particle size, surface charge, encapsulation efficiency percent, and 24 h cumulative drug release for increasing the effectiveness and selectivity of FLT against prostate cancer. Moreover, in vitro cytotoxicity assay, ex vivo permeation through rabbit intestine, and in vivo pharmacokinetic study will be performed comparing the optimized FLT-loaded niosomes and the free drug suspension in order to confirm and validate the enhancement of efficacy, drug absorption, and bioavailability.

## 2. Materials and Methods

### 2.1. Materials

Flutamide was obtained as a gift from Sigma for Pharmaceutical Industries (Qwesna, Egypt). Sorbitan monostearate (Span 60) was purchased from Merck Schuchardt OHG (Hohenbrunn, Germany). Cholesterol from lanolin, dicetyl phosphate (DCP), dimethyl sulfoxide, and 3-(4,5-dimethylthiazol-2-yl)-2 and 5-diphenyltetrazolium bromide salt (MTT) were obtained from Sigma-Aldrich Company (St. Louis, MO, USA). Chloroform HPLC was purchased from Central Drug House Ltd. (New Delhi, India). Absolute HPLC methanol was purchased from VWR International (Paris, France). Deionized water, potassium phosphate dibasic anhydrous (K_2_HPO_4_), Polysorbate 80, sodium azide and glass beads were obtained from Loba chemie (Mumbai, India). Orthophosphoric acid was obtained from Biochem (Cairo, Egypt).

### 2.2. Experimental Design

Based on the literature, the two main factors affecting niosomal formulation are cholesterol and nonionic surfactants in addition to DCP as a negative surface charge-inducer to lower the probability of aggregation and increase formulation stability. [37]. A three-level four-factor D-LSCD design was used for statistical optimization of the formulation variables for preparing FLT-loaded niosomes. The four formulation factors are selected to be Span 60 concentration (X_1_), cholesterol concentration (X_2_), DCP concentration (X_3_) and drug concentration (X_4_) to study their effect on the selected responses where the objective is to minimize mean particle size (Y_1_) and maximize the remaining dependent variables: zeta potential (Y_2_), encapsulation efficiency percent (Y_3_) and cumulative drug release after 24 h (Y_4_). Independent variables with their levels and responses are shown in Table 1. Eighteen experimental runs were generated, with (eight runs as the cube points, eight runs as the star points, and two runs as the midpoint). For every factor, two axial points were selected to be 1.68 at the upper and lower ultimate levels in order to make the design rotatable, with randomization of the runs to eliminate the block effects. The components of the formulated FLT-loaded niosomes according to D-LSCD are revealed in Table 2. The design was generated, and mathematical relations were elucidated as multinomial equations via using the statistical software Statgraphics^®^ Centurion XV, version 15.2.05 (StatPoint, Inc., Warrenton, VA, USA). The significance of multinomial equations was explained by ANOVA.

### 2.3. Formulation of FLT-Loaded Niosomes

Thin-film followed by hydration technique was used to formulate FLT-loaded and drug-free niosomes with slight variations [38,39]. Initially, a blend of 100 mg of both cholesterol and Span 60 were precisely weighed according to their molar ratios, afterward, a different amounts of DCP and FLT were also added to the aforementioned mixture in long-necked pear-shaped flask and dissolved in 25 mL of (1:5) chloroform: methanol solution [40,41]. Then the flask is allowed to rotate on a water bath maintained at 55 ± 2 °C under reduced pressure using a rotatory vacuum evaporator, Büchi-M/HB-140, (Flawil, St. Gallen, Switzerland) operated at 50 RPM for 30 min which allows slow and complete evaporation of the organic solvents leaving a thin dry layer of surfactant and cholesterol mixture left on the inner wall of the rounded flask. Then hydration of the dry film was done via the addition of 10 mL deionized water in the presence of 10 glass beads of 4 mm diameter and rotation of the flask in the previously mentioned conditions for 20 min to ensure complete hydration [38,40]. Finally, the suspension was vortexed for 10 min, sonicated at 60 °C for 15 min at 20 kHz and refrigerated at −20 °C till further analysis [42].

### 2.4. Separation and Washing of FLT-Loaded Niosomes

The iced-up FLT niosomal dispersion was defrosted beyond the preparation temperature viz., 55 °C, as it is stated that the encapsulation of the drugs into niosomes was greatly enhanced by freeze-thawing. Separation of unencapsulated FLT from the niosomal suspension was done by cooling centrifuge (Centurion Scientific Ltd., Stoughton, UK) at the force of 10,000× *g* and temperature of about 2 °C. After removal of the supernatant, niosomal pellets were rinsed in deionized water and centrifuged once more after re-dispersion with a vortex mixer. To ensure that the free FLT (un-entrapped) was no longer present in the spaces between the niosomes, the washing procedure was done twice [43].

### 2.5. Encapsulation Efficiency (EE) Determination

The direct method of determination of encapsulation efficiency was utilized as follows, 250 μL of the washed niosomal suspension was accurately drawn using 100–1000 μL Micropipette, Dragon lab scientific Co., Ltd. (Beijing, China) then completed to 10 mL volume using methanol which serves to burst the formed vesicles and release the entrapped drug followed by sonication till the clear solution was obtained that detected spectrophotometrically at wavelength 304 nm [14] using UV-visible spectrophotometer, Jasco V-630 (Tokyo, Japan). Drug-free niosomes treated with the same technique were used as a blank in the measurement. All measurement were repeated three times then the EE% was calculated using Equation (1) as follow [44,45].

(1) $$\mathrm{EE}\%=\frac{\mathrm{Amount}\;\mathrm{of}\;\mathrm{entrapped}\;\mathrm{FLT}}{\mathrm{Total}\;\mathrm{amount}\;\mathrm{of}\;\mathrm{FLT}\;\mathrm{added}\;\mathrm{in}\;\mathrm{the}\;\mathrm{formulation}}\times 100$$

### 2.6. Characterization of FLT-Loaded Niosomes

#### 2.6.1. Measurement of the Mean Particle Size (PS) and Zeta Potential (ZP)

Prior to determination all formulations were diluted to a suitable strength with deionized water and sonicated for 5 min to eliminate air and break down any clumps of particles [34,46]. Then the mean nanoparticle diameter expressed in nm, surface charge expressed as mV and polydispersity index (PDI) for all prepared FLT-loaded niosomal formulations at 25 °C using Quasi-elastic light scattering (QELS) based on laser diffraction (NICOMP^TM^ 380 ZLS NICOMP particle sizing system, Santa Barbara, CA, USA) equipped with a 5-mW laser with a wavelength output of 632.8 nm. All measurements were repeated three times and the results were displayed as average value ± standard deviation.

#### 2.6.2. In Vitro Release Study of FLT-Loaded Niosomes

The dialysis bag diffusion method was utilized to study in vitro release pattern of FLT from the niosomal suspension [47,48], a constant volume (3 mL) from each formulation (F1-F18) was wrapped into a dialysis bag (VISKING^®^ Dialysis Tubing MWCO12,000–14,000) with 4 cm length and 2.1 cm width (prior to experimentation day all dialysis bags were soaked for one night in the release medium to permit excellent diffusion). After that, the bags were immersed in 50 mL phosphate buffer solution (PBS) containing 0.2% Tween 80 (to enhance solubilization of FLT and achieve the sink condition) and 0.02% sodium azide (as a preservative) [49], the solution pH was adjusted at 7.4 using anhydrous K_2_HPO_4_ and orthophosphoric acid. The release was conducted using an incubator shaking benchtop (ThermoStable^TM^ IS-20, Daihan Scientific Co, Ltd., Seoul, Korea). The shaker was operated at 140 rpm and maintained at 37 °C. Aliquots of 1 mL were withdrawn and immediately replenished with fresh medium at designated intervals of 0.5, 1, 2, 4, 6, 8, 12 and 24 h. The withdrawn samples were analyzed with a UV spectrophotometer at 304 nm wavelength to measure the amount of the drug in the release medium. The results were expressed as a percentage of cumulative drug release over 24 h.

#### 2.6.3. Mathematical Modeling of Flutamide Release from Niosomal Formulations

The data of the release study were mathematically tailored to the release kinetic models (zero, first, second-order, Higuchi diffusion, Baker–Lonsdale, Hixon–Crowell, and Korsmeyer–Peppas release) by comparison of the correlation coefficients (r) where the model with the highest coefficient was selected to be the best-fitting model.

### 2.7. Prediction, Formulation and Evaluation of the Optimized Formula

D-LSCD was effectively implemented, and all trials were applied by selecting the dependent and independent factors with the indicated levels. The obtained results for each response (Y_1_–Y_4_) were analyzed and after the manifold response optimization the optimized FLT formula was predicted then prepared and assessed three times for all responses (Y_1_–Y_4_) to check the authenticity of the measured optimized formula responses and the predicted responses. Moreover, the optimized formula was subjected to further experiments to prove its pharmacokinetic and cytotoxic effects.

### 2.8. Transmission Electron Microscopy

A transmission electron microscope (Jeol: JEM-2100, Tokyo, Japan) was used in order to affirm the formation of the double-layered structure of the niosomes and determine their size precisely. The optimized formula (OF) was greatly diluted with deionized water to suitable intensity to allow a clear vision of the formed niosomes. One drop of the diluted suspension was spread onto a grid coated with carbon and left for one minute to permit some of the particles to attach to the carbon substrate. The surplus dispersion was then removed with a piece of filter paper. After that, a drop of 1% solution of phosphotungstic acid was added as a staining solution and filter paper was used to remove the extra staining solution. Finally, the sample was allowed to dry in the open air before being inspected under an electron microscope [50,51].

### 2.9. In Vitro Cytotoxicity Study

Optimized FLT-loaded niosomal formulation was compared with free FLT suspension for their in vitro cytotoxicity against prostate cancer cell line (PC-3), human breast adenocarcinoma (MCF-7) and normal cells of green monkey epithelial cells (VERO). IC_50_ values obtained from different treatments were measured by using GraphPad Prism version 5 software (GraphPad Software Inc., San Diego, CA, USA).

#### 2.9.1. Cell Culture

Cancer cells from different cancer cell lines (MCF7, PC-3 and normal cell line (VERO)) were purchased from American Type Culture Collection (Manassas, VA, USA) and grown on Roswell Park Memorial Institute medium (RPMI 1640) supplemented with 1% of 100 mg/mL streptomycin, 100 units/mL of penicillin and 10% of heat-inactivated fetal bovine serum in a humidified, 5% (*v/v*) CO_2_ atmosphere at 37 °C.

#### 2.9.2. Cytotoxicity Assay (MTT)

Exponentially growing cells from different cell lines were trypsinized, counted and seeded at the appropriate densities (5000 cells/0.33 cm^2^ well) into 96-well microtiter plates. Cells were then incubated in a humidified atmosphere at 37 °C for 24 h. Then, cells were exposed to different concentrations of blank niosomes, free FLT suspension and optimized FLT formula (0.1, 10, 100, 1000 µg/mL) for 48 h. Then the viability of treated cells was determined using MTT technique. Media were removed and the cells were incubated with 200 μL of 5% MTT solution/well (Sigma Aldrich, MO, USA). Cells were then allowed to metabolize the dye into a colored-insoluble formazan crystal for 2 h and the formazan crystals were dissolved in 200 µL/well DMSO. Absorbance was measured at 570 nm using Epoch-2c plate reader (BioTeck, Winooski, VT, USA). The cell viability was expressed as a percentage of control and the concentration that induces 50% of maximum inhibition of cell proliferation (IC_50_) was determined using GraphPad Prism version 5 software (GraphPad Software Inc., San Diego, CA, USA) [52,53].

### 2.10. Ex Vivo Permeation Study through Rabbit Duodenum

#### 2.10.1. Protocol

The small intestine of rabbits was used to test the enhancement in the duodenal permeability of optimized FLT-loaded niosomes compared to free FLT suspension. The duodenum of the small intestine was dissected into 1 cm parts and cleaned with Ringer’s solution to remove mucus and lumen. Then duodenum’s one end was ligated with thread and filled with 1 mL of tested sample (equivalent to 0.66 mg drug) using 100–1000 μL micropipette, followed by tying the other end tightly with thread as shown in Figure 6A. After that, the closed duodenum filled with the tested sample was immersed in a 50 mL phosphate buffer solution of pH 7.4 containing 0.2% Tween 80 and 0.02% sodium azide at 37 °C with constant stirring (140 rpm) in a shaking incubator [54].

#### 2.10.2. Samples Collection and Analysis

Aliquots (1 mL) were withdrawn at predetermined intervals (0.25, 0.5, 1, 2, 4, 6, 8, and 12 h) and replenished immediately with a fresh medium. The aliquots were analyzed using HPLC at 304 nm to calculate FLT concentration in all samples based on the calibration curve of the drug in the release medium, results were expressed as % of cumulative drug permeated over 12 h. The trial was done in triplicate.

#### 2.10.3. Permeation Data Analysis

The accumulative amount of FLT permeated (Q) was plotted against time. The steady-state flux (J_ss_) was calculated from the slope of the linear portion of the accumulative amount permeated per unit area versus time plot. The permeability coefficient (PC) of the drug through the intestine was calculated by dividing steady-state flux with the initial concentration of FLT. The enhancement ratio (ER) was calculated by using the equation [55]:

(2) $$\mathrm{ER}=\frac{J_{\mathrm{ss}}\;\mathrm{of}\;\mathrm{optimized}\;\mathrm{Flutamide}\;\mathrm{niosomes}}{J_{\mathrm{ss}}\;\mathrm{of}\;\mathrm{free}\;\mathrm{Flutamide}\;\mathrm{suspension}}$$

### 2.11. In Vivo Pharmacokinetic Study of the Optimized Formula on Male Rats

#### 2.11.1. Protocol

Animal study was executed in accordance with the protocol approved by the Animal Ethical committee approval of Faculty of Pharmacy, Cairo University (No. PI 2846) using the Sprague-Dawley male rats with mean weight (250 ± 20 g) housed in plastic mesh cages under normal conditions of light (12 h light/dark rotations), relative humidity and temperature of 25 °C and fed with the standard laboratory diet and water during the study. First, the rats were distributed into two groups of six rats each (X and Y). Then, one day before the experiment, all rats weren’t allowed to access food with open access to water overnight. Finally on the day of the experiment both groups X (test group) and Y (control group) were given a single oral dose of the optimized FLT-loaded niosomes and free drug suspension (26 mg/kg), respectively [14]. Free drug suspension was prepared using 0.2% gum tragacanth and glycerin in deionized water [34].

#### 2.11.2. Samples Collection and Storage

Blood samples (1.5 mL) were collected at predetermined intervals (0 “predose”, 10, 15, 20, 30, 45, 60, 90, 120, 180, 240, 300, 360, 480, 720, and 1400 min) from retro-orbital plexus in screw-top EDTA spiked tubes. Samples were centrifuged at 6000 rpm for 10 min using a cooling centrifuge K241R (Centurion Scientific Ltd., Stoughton, UK) to separate the plasma of the blood that was frozen at −80 °C using an Ultra-Low temperature freezer (WUF-25, Daihan Scientific Co, Ltd., Seoul, Korea) till further HPLC analysis using the previously described HPLC method [14,56,57].

#### 2.11.3. Plasma Samples Treatment and HPLC Assay

Aliquots of plasma (0.75 mL) were treated with methanol in a 1:2 ratio, mixed for 1 min using vortex mixer (Paramix II, Julabo Labortechnik GmbH, Seelbak, Germany), and then centrifuged at 15,000 rpm for 12 min to separate the plasma denatured proteins. An amount of 100 μL of the clear supernatant was injected into the HPLC column for the analysis of FLT plasma concentration. A reverse phase HPLC method was used for the assay of FLT. The analysis was carried out using HPLC (Waters alliance 2695, Milford, MA, USA) occupied by RP-18, 250 × 4.6 mm column (Xterra, Milford, MA, USA) and PDA detector. An isocratic system consisting of methyl alcohol: water in ratio 75:25 (*v/v*) was used at a flow rate of 1 mL/min and an injection volume of 100 μL and the peaks were detected at 304 nm. Under these conditions, the total run time was about 7 min, and the retention time was approximately 5 min. Calibration curves (peak area versus concentration) were linear (R^2^ > 0.998) over the FLT concentration range of 0.25–10 μg/mL. The different pharmacokinetics parameters were calculated using the non-compartmental method by PK Solver 2.0 software (an add-in program for pharmacokinetic data).

## 3. Results and Discussion

In our work, 18 formulae of FLT niosomes were prepared as recommended by D-LSCD. FLT encapsulation in niosomal drug transporter system aimed to enhance solubility and the oral bioavailability of encapsulated FLT.

### 3.1. Experimental Design (D-LSCD)

Among the well-known experimental designs are Box–Behnken design (BBD) and central composite design (CCD). Concerning the data mentioned in Table 1, our study involves four independent variables (X_1_–X_4_). In this case, both CCD and BBD generate 30 runs and 27 runs respectively so D-LSCD was utilized as a highly proficient statistical design in order to shrink the total number of runs and upsurge the efficiency, for four factors, the quadratic equations (Equations (3)–(6)) involve 15 coefficients and the total runs were solely 18 (Table 2).

### 3.2. Response Surface Methodology (RSM) for the Optimization of FLT-Loaded Niosomes

**Figure 1:**
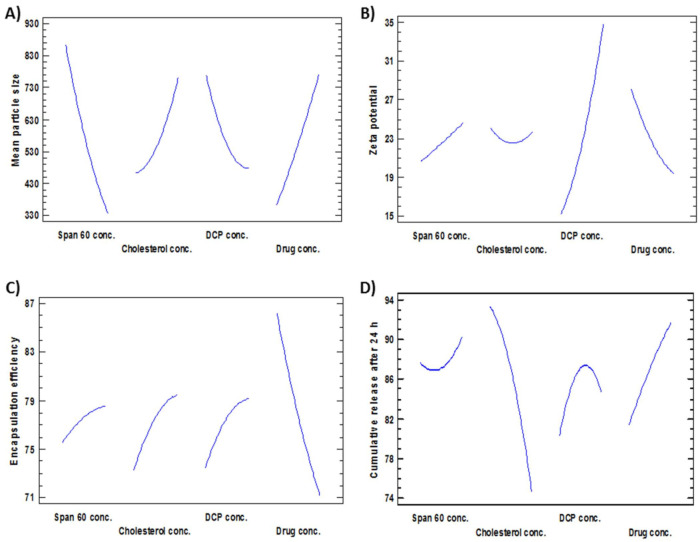
Main effects plots revealing the effect of the investigated factors (X_1_–X_4_) on (**A**) Mean particle size, (**B**) Zeta potential, (**C**) Encapsulation efficiency and (**D**) Cumulative release after 24 h.

**Figure 2:**
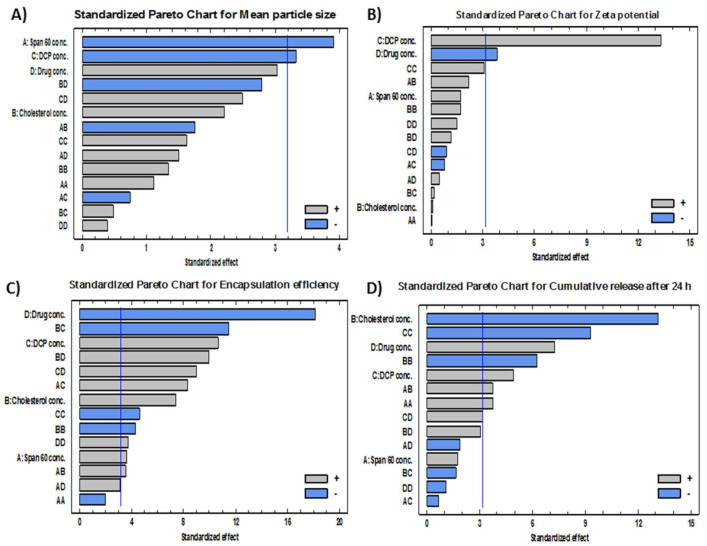
Standardized Pareto charts showing the effects of the investigated factors (X_1_–X_4_) on (**A**) mean particle size, (**B**) zeta potential, (**C**) encapsulation efficiency and (**D**) cumulative release after 24 h.

**Figure 3:**
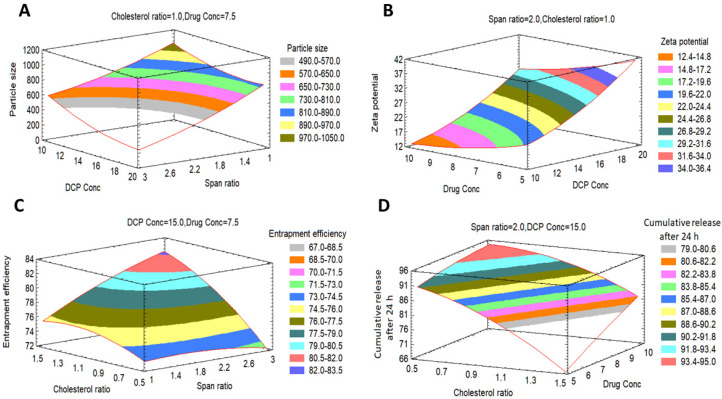
Expected response surfaces with 3D contour plots showing the effects of the investigated factors (X_1_–X_4_) on (**A**) mean particle size, (**B**) zeta potential, (**C**) encapsulation efficiency and (**D**) cumulative release after 24 h.

To analyze the D-LSCD formulations statistically, manifold regression analysis with the Statgraphics program and two-way ANOVA as statistical tests were utilized. The assessed factor effects and related *p*-values for the 4 factors from ANOVA were shown in Table 3 where a positive sign indicates synergistic outcome (direct relationship between the factor effect and the examined response), while a negative sign indicates an antagonistic outcome (inverse association between the factor effect and the examined response), the factor effect is considered significant if the effect differs from zero and *p*-value is lower than 0.05. Figure 1 revealed the factors which have the main effect on each response. Additionally, Pareto charts in Figure 2 demonstrated the correlation between the factors and the responses and their significant ones. Furthermore, 3D plots (response surface) in Figure 3 showed the effect of all factors on the responses over the nominated levels of factor.

#### 3.2.1. Estimation of the Quantitative Effects of the Factors

##### Effects on the Mean Particle Size (Y_1_)

The mean particle size of all formulations (Y_1_) ranged from 179 nm for F4 to 1293 nm for F16 as shown in Table 2. The variance of all formulations ranged from 0.035 for F11 to 0.68 for F17 that indicates an even distribution of particle size. Figure 1A, Figure 2A and Figure 3A revealed that concentration of Span 60 (X_1_) was the major factor responsible for the difference in mean particle size of all prepared FLT-niosomal formulae. It was observed that X_1_ has a significant inverse relationship on the particle size Y_1_ with a *p*-value of 0.0298. An example for the effect of Span 60 on the particle size was the change in the particle size between F10, F2 and F6 where; at constant level of X_2_, X_3_ and X_4_, an increase in the ratio of Span 60 from 0.318 M to 3.682 M will lead to decrease in the Y_1_ from 1087.9 nm to 204.8 nm. This outcome was in agreement with the results achieved in a previous study for the effect of Span 60 amounts on the mean particle size [58] that may be due to Span 60 enhancing solubilization of the hydrophobic drugs that lead to reduction of particle size [59]. Additionally, the amount of DCP added (X_3_) had a significant inverse effect on Y_1_ with *p*-value of 0.0450, this indicated by the difference in vesicle size between F18 and F13 where; at the same level of X_1_, X_2_ and X_4_ a rise in the amount of DCP from 6.59 mg to 15 mg led to reduction in the mean particle size from 1063.9 nm to 600.6 nm. These outcomes could be elucidated by the neutralizing influence of DCP for the positively charged drug and so decreasing the likelihood for aggregation so, reducing the particle size [60,61]. The equation of the model is:

Y_1_ = 1779.53 − 434.615 X_1_ + 1720.55 X_2_ − 197.4 X_3_ − 40.653 X_4_ + 52.113 X_1_^2^ − 306.942 X_1_X_2_ − 8.448 X_1_X_3_ + 52.969 X_1_X_4_ + 249.745 X_2_^2^ + 10.875 X_2_X_3_ − 196.288 X_2_X_4_ + 3.012 X_3_^2^ + 11.241 X_3_X_4_ + 2.922 X_4_^2^ (3)

##### Effects on Zeta Potential (Y_2_)

ζ potential of all preparations (Y_2_) was ranged from −10.46 mV for F9 to −46.76 for F15, as shown in Table 2. Figure 1B, Figure 2B and Figure 3B confirmed that DCP concentration (X_3_) was the most important factor responsible for the difference in ζ potential of FLT-loaded niosomes as it has a significant direct effect on the ζ potential (Y_2_) with a *p*-value of 0.0009. For instance, an increase in the amount of DCP added from 10 mg to 20 mg at unchanged levels of X_1_, X_2_ and X_4_ led to the increase (increasing the value with ignoring the negative sign) in ζ potential from −26.06 mV to −46.76 mV for F7 and F15, respectively. The same conclusion was noted in F2 or its twin F13 and F5 by increasing the ζ potential from −23.45 mV to −46.74 mV with increasing DCP amount from 15 mg to 23.4 mg, respectively. This theory can be attributed to the structure of FLT which carry a positive charge on the quaternary amine group that has been counteracted by DCP addition, this finding also lead to a significant antagonistic effect between the ζ potential and the amount of drug added with *p*-value of 0.0315, via observing F3, F2 and F12, at the same level of the other factors, on the rise of drug amount from 3.29552 mg, 7.5 mg and 11.7045 mg, a reduction in ζ potential from −32.37 mV, −23.45 mV to −17.79 mV, respectively. The mathematical equation of the model is:

Y_2_ = 58.189 − 4.681 X_1_ − 35.882 X_2_ − 0.269 X_3_ − 5.585 X_4_ + 0.059 X_1_^2^ + 6.508 X_1_X_2_ − 0.152 X_1_X_3_ + 0.292 X_1_X_4_ + 5.468 X_2_^2^ + 0.084 X_2_X_3_ + 1.393 X_2_X_4_ + 0.098 X_3_^2^ − 0.068 X_3_X_4_ + 0.192 X_4_^2^ (4)

##### Effects on the Encapsulation Efficiency% (EE%) (Y_3_)

The EE% of all formulations (Y_3_) was in the range of 48.37% for F14 to 93.34% for F3 as shown in Table 2. Figure 1C, Figure 2C and Figure 3C revealed that the most significant factor that inversely affects the EE% was X_4_ (drug amount) with a *p*-value of 0.0004, when X_1_, X_2,_ and X_3_ are constant; an increase in the drug amount from 3.29 mg to 7.5 mg decreases the EE% from 93.3% to 77.2% for F3 and F2, respectively this may be as a result of saturation of the double layers of the formed niosomes at a certain level of drug incorporation so that a further increase in the drug amount will lead to decreasing EE% and that is confirmed by the presence of drug crystals in between the formed niosomes under the optical microscope for example in both F3 and F12 [62]. Additionally, all the other factors X_1_, X_2,_ and X_3_ were directly affecting the EE% with *p*-values of 0.0359, 0.0050 and 0.0018 respectively. Firstly, regarding X_1_ (Span 60 concentration) by increasing amount from 0.318 M in F10 to 3.68 M in F6 with all other factors held constant, the EE% augmented from 73.5% to 78.6% respectively this may be credited to increase the accommodation of the drug molecules within the available lipophilic environment created by the increased Span 60 concentration [63]. Secondly concerning X_2_ (cholesterol concentration), when X_1_, X_3_ and X_4_ are constant; an increase cholesterol concentration from 0.159 M in F1 to 1 M in F2 increases the EE% from 69% to 77% respectively also seen in F13 and F8 where increasing cholesterol concentration from 1 M to 1.84 M will increase the EE% from 77.7% to 79.3% respectively. This can be explained by the cholesterol ability to cement the leaky space in the bilayer membranes, which in turn prevent escaping of the drug from the formed niosomes and so enhancing the EE% [63]. Finally at an equal level of X_1_, X_2_ and X_4_, increasing the DCP amount from 6.59 mg to 23.4 mg will increase EE% from 68.3% to 79.5% for F18 and F5 respectively, also increasing the DCP amount from 10 mg to 20 mg will increase EE% from 86.9% to 87.8% for F7 and F15 respectively. The attraction forces between negatively charged DCP and the positively charged drug may be a good explanation of this finding [37]. The mathematical equation that best fits the model is:

Y_3_ = 144.539 − 13.772 X_1_ − 0.1182 X_2_ + 0.725 X_3_ − 14.972 X_4_ − 0.576 X_1_^2^ + 3.863 X_1_X_2_ + 0.575 X_1_X_3_ + 0.678 X_1_X_4_ − 4.956 X_2_^2^ − 1.589 X_2_X_3_ + 4.305 X_2_X_4_ − 0.053 X_3_^2^ + 0.249 X_3_X_4_ + 0.173 X_4_^2^ (5)

##### Effects on the Cumulative FLT Release after 24 h (Y_4_)

**Figure 4:**
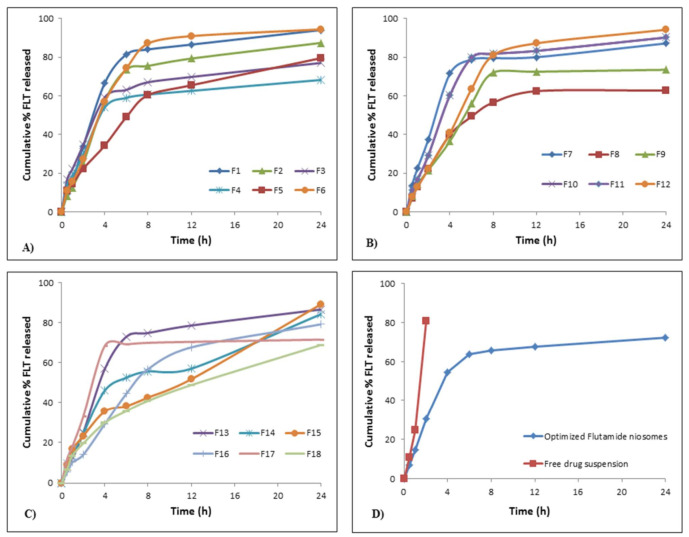
In vitro flutamide release profiles; (**A**) release profiles for F1–F6, (**B**) release profiles for F7–F12, (**C**) release profiles for F13–F18, (**D**) release profiles for optimized FLT-loaded niosomes against free FLT suspension.

FLT-loaded niosomes showed variation in the cumulative FLT release after 24 h (Y_4_) ranged from 62.7% for F8 to 94.2% for F12 as shown in Table 2. Figure 1D, Figure 2D and Figure 3D clearly revealed that the main significant factor that inversely affecting niosomal FLT release was factor X_2_ (cholesterol concentration) with a *p*-value of 0.001. The previous note confirmed via fixing all other factors X_1_, X_3_ and X_4_ then changing cholesterol concentration from 0.159 M for F1, 1 M for F2 to 1.84 M for F8 will decrease release from 94%, 87.3%, 62.7% respectively as depicted in Figure 4A–C. This can be attributed to the stabilizing effect of the cholesterol to the niosomal bilayers which, prevents drug leakage, and so delays the efflux of the drug enclosed within the niosomes [64]. The other 2 significant factors that directly proportionate with the cumulative drug release after 24 h were the amount of DCP and drug added with *p*-values 0.0163 and 0.0055 respectively as shown in Table 3. Firstly, as noted from Table 2; increasing DCP amount from 6.59 mg for F18 to 23.4 mg for F5 will enhance the release from 68.8% to 79.5%, the same finding for F7 and F15 where increasing DCP amount from 10 mg to 20 mg will increase release from 87.1% to 89.3% respectively. This could be explained by the enhanced separation on the double-layered structure of the niosomes caused by DCP [65]. Furthermore, increasing drug amount from 3.29 mg for F3 to 11.7 mg for F12 will increase release from 77% to 94.2% respectively. This may be due to the increased amount of free drug crystals between the formed niosomes by the increasing amount of drug added. The equation that best fits the model is:

Y_4_ = 59.452 − 6.576 X_1_ − 18.783 X_2_ + 5.332 X_3_ + 0.174 X_4_ + 1.829 X_1_^2^ + 6.921 X_1_X_2_ − 0.079 X_1_X_3_ − 0.698 X_1_X_4_ − 12.343 X_2_^2^ − 0.391 X_2_X_3_ + 2.252 X_2_X_4_ − 0.183 X_3_^2^ + 0.152 X_3_X_4_ − 0.085 X_4_^2^ (6)

##### Kinetic Behavior of FLT Release from the Prepared Niosomes

As depicted in Figure 4A–C, release of FLT from nearly all formulae (F1–F18) followed a biphasic behavior; an early fast release phase that took about 2–3 h followed by a sustained plateau phase that was maintained for 24 h. The initial phase may be explained by the release of the surface adsorbed drug on niosomal formulae while; the sustained plateau phase occurs as a result of the diffusion mechanism through the bilayers of niosomal vesicles [66]. This is confirmed by the drug release kinetics for the 18 formulations shown in Table 4 which reveals that the highest correlation coefficient (r) values for all formulations except for F1, F2, F6, F10, F12 and F13 were followed by diffusion and Baker–Lonsdale release models. This conclusion approves the fact that niosomes can act as a drug reservoir for sustained drug delivery and these results were similar to previous several studies [67,68]. Additionally, this sustained release pattern of drug entrapped might refer to the high stability of the designed system [41].

#### 3.2.2. Estimation of the Quantitative Effects of Quadratic Term and Interactions

Firstly concerning the quadratic terms, it was noted that X_1_^2^ had a significant synergistic effect on the cumulative drug release percentage after 24 h (Y_4_) with a *p*-value of 0.0337; whereas X_2_^2^ and X_3_^2^ had a significant antagonistic effect on both the encapsulation efficiency (Y_3_) with *p*-values of 0.0228, 0.0191 and cumulative drug release percentage after 24 h (Y_4_) with *p*-values of 0.0081, 0.0026 respectively, it was also observed that X_4_^2^ had a significant synergistic effect on EE% (Y_3_) with *p*-values of 0.0328. On the other hand about the second-order interactions, it was concluded that X_1_X_2_, X_1_X_3_, X_2_X_4_, X_3_X_4_ had a significant synergistic effect on EE% (Y_3_) with *p*-values of 0.0373, 0.0037, 0.0021 and 0.0029 respectively, while the interaction X_2_X_3_ had a significant antagonistic effect on EE% (Y_3_) with a *p*-value of 0.0014; X_1_X_2_ and X_3_X_4_ had a significant synergistic effect on the cumulative drug release percentage after 24 h (Y_4_) with *p*-values of 0.0333 and 0.0494, respectively.

### 3.3. Preparation of the Optimized FLT-Loaded Niosomal Formula

An optimized FLT niosomal formulation with minimal vesicular particle size and maximum zeta potential value, encapsulation efficiency percent and 24 h cumulative release percent was successfully obtained through D-LSCD. In order to obtain a mixture of factor levels that augment the desirability function, the final optimized parameters were considered and analyzed to compromise among different responses. The reliability of the D-LSCD results was authenticated by preparing a new formulation according to the expected model and evaluated for the responses as listed in Table 5. The optimized formula was prepared by the gained optimal values of variables which were 1.00 M, 0.93 M, 23.08 mg and 6.66 mg of X_1_, X_2_, X_3_ and X_4_ respectively using thin-film hydration procedure.

### 3.4. Characterization of the Optimized FLT-Loaded Niosomes

#### 3.4.1. EE%, Particle Size, ζ Potential and Cumulative FLT Release after 24 h

As shown in Table 5; the observed values of the responses were compared with the predicted values as follow; the observed responses values for Y_1_, Y_2,_ Y_3_ and Y_4_ were found to be 748.6 nm, −48.60 mV, 72.8% and 72.2%, respectively, whereas the predicted values were 736.4 nm, −46.76 mV, 74.5% and 78.6%, respectively. The comparison revealed no considerable residuals, and the predicted error percentage of the responses was 8%, indicating that the working design was reasonably valuable for optimizing FLT-loaded niosomes.

#### 3.4.2. In Vitro FLT Release

As depicted in Figure 4D, the in vitro FLT release is done as mentioned previously to compare the pattern of release of both FLT-loaded niosomes and free FLT suspension. It was observed that more than 80% of free drug suspension was released within the first 2 h, where the optimized niosomes show sustained drug release over more than 12 h in addition to that the release pattern of the free FLT suspension was zero-order whereas the release pattern from optimized FLT-loaded niosomes were by diffusion mechanism as shown in Table 4.

#### 3.4.3. Transmission Electron Microscopy

**Figure 5:**
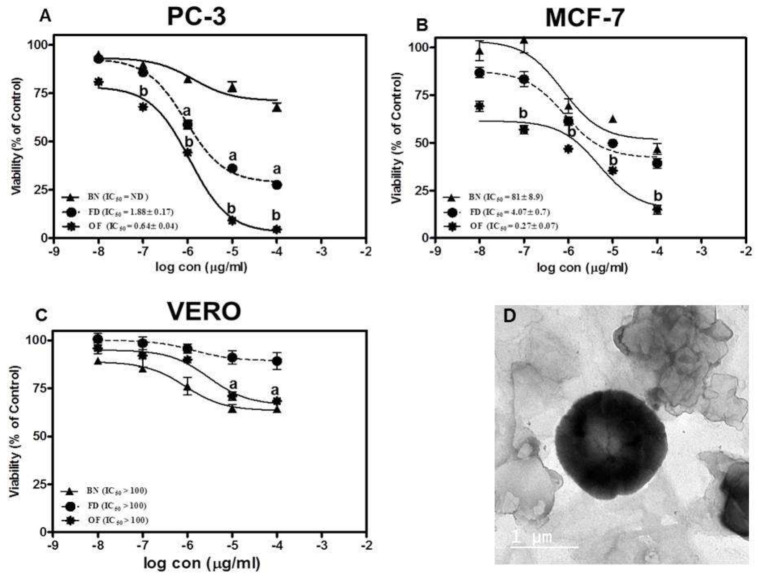
Cytotoxicity assay against (**A**) prostate cancer cell line (PC-3), (**B**) breast adenocarcinoma (MCF-7) and (**C**) normal hamster kidney cell line (VERO) where a. denotes significant from blank niosomes (BN) at *p* < 0.01, b. denotes significant from BN and free drug (FD) *p* < 0.01. (**D**) TEM image of the optimized FLT-loaded niosomes.

The vesicular morphology was examined using a transmission electron microscope (TEM). The micrographs confirmed the formation of niosomal vesicles with distinctive bilayer structure. The vesicle core was clearly observed with its sphere-shaped structure in the photomicrograph was revealed in Figure 5D.

### 3.5. In Vitro Cytotoxicity of the Optimized FLT-Loaded Niosomes

The cytotoxicity of the optimized FLT-loaded niosomes (OF) was performed using MTT assay against PC-3 (Prostate cancer cell lines), MCF-7 (Brest adenocarcinoma) and VERO (green monkey epithelial kidney cells) compared to free FLT suspension. The results were presented in Figure 5A,B which shows that the drug-loaded niosomes induced three-fold reduction in the IC_50_ to 0.64 ± 0.04 µg/mL compared to 1.88 ± 0.16 µg/mL for FLT treatment on PC-3 (*p* < 0.01). Moreover, treatment of MCF-7 with optimized FLT niosomes showed 15 folds reduction in the IC_50_ compared to free FLT suspension with IC_50_ of 0.27 ± 0.07 µg/mL and 4.07 ± 0.74 µg/mL respectively. Finally, it can be concluded that niosomes significantly increased the effect of FLT on cancer cells probably through an increase in the intracellular concentration and cellular uptake with the common basics and usual characteristics of nanoparticles such as niosomes. This style of cell-nanoparticle interaction was often accompanied by internalization via fast non-specific phagocytosis [69]. Nano-carriers have numerous benefits over free drugs, as protection from breakdown, selective and improved absorption into the targeted tissue, and regulate the pharmacokinetics and drug tissue distribution profile. These consequences were in good agreement with prior studies on many nanoparticles and niosomes that exhibited that the effectiveness of the drug could be enhanced by niosomal encapsulation and the amount of the drug to be used can be decreased and so the safety will sequentially be also improved [70,71,72,73,74,75].

In terms of safety for the normal cells, cytotoxic effects of free FLT and optimized FLT-loaded niosomes were evaluated against VERO cells over identical concentration range (from 0.01 to 100 µg/mL) where both exerted a non-cytotoxic effect on the VERO cells with concentration >100 µg/mL as shown in Figure 5C. That proves the selectivity of the drug towards the cancer cells only without significant harm to the normal body cells.

### 3.6. Ex Vivo Permeation Study through Rabbit Duodenum

**Figure 6:**
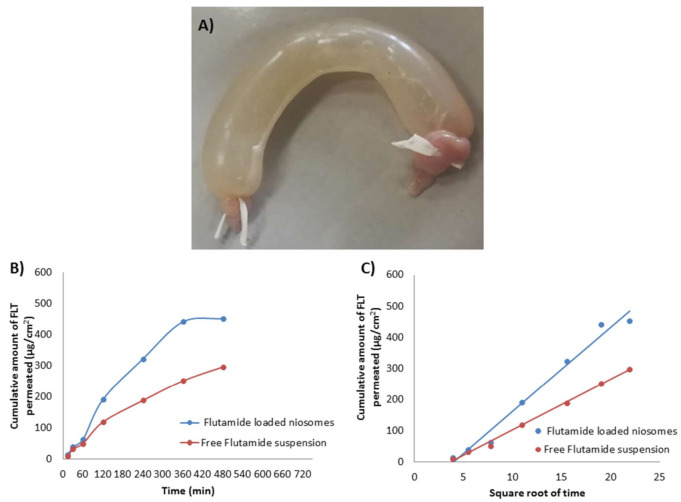
(**A**) Freshly excised and ligated rabbit intestine filled with niosomes, (**B**) Cumulative amount of FLT released against time and (**C**) Cumulative amount of FLT released against $\sqrt{t}$.

Ex vivo intestinal permeation study of FLT-loaded niosomes and free FLT suspension was examined via freshly excised rabbit intestine. The FLT permeation profile Figure 6B,C demonstrate that niosomes permeate more than free drug with about 1.5 times increase in the intestinal absorption reflected by a steady-state flux of 0.751 and 0.495 μg/cm^2^/min for FLT-loaded niosomes and free FLT suspension respectively, also by permeability coefficient of 1.139 and 0.750 μg/min × 10^−3^ for FLT-loaded niosomes and free FLT suspension respectively. The previous results show significance at *p*-value < 0.05, implying a role for vesicles, which could be due to their large surface area for interactions. These improvements in FLT transport in niosomes could be attributable to the formulation’s particle size, high permeability across the intestine, and drug release profile characteristics. The enhancement in drug permeation mechanism by niosomes may also be guessed because niosomes interact thermodynamically with the barriers as the membrane of the intestine so enhancing the permeability across them which leads to improving the bioavailability [76].

### 3.7. In Vivo Pharmacokinetic Study of the Optimized Formula on Male Rats

**Figure 7:**
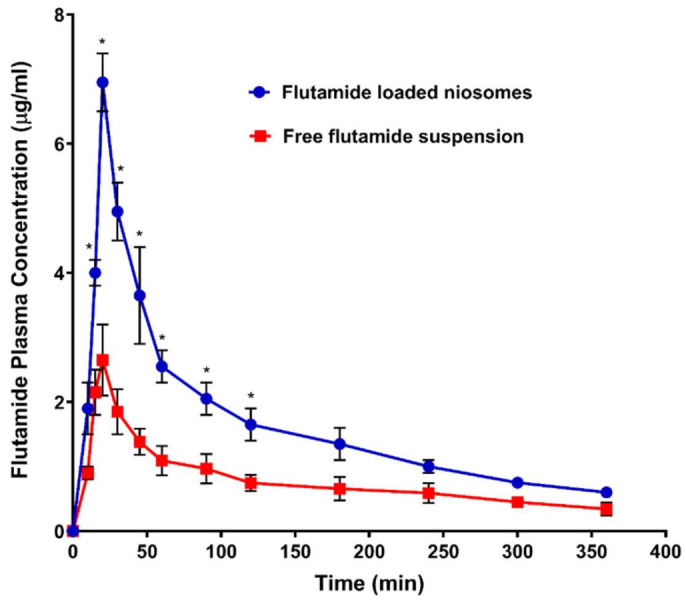
Plasma concentration of flutamide (FLT) after oral administration 26 mg/kg of both FLT-loaded niosomes and free FLT suspension. Note: * Significant effect at *p*-value < 0.05 at a specified time point.

Figure 7 and Table 6 showed the mean plasma concentration-time curve and the calculated pharmacokinetic parameters of FLT respectively after oral single-dose administration of either FLT-loaded niosomes or free Flutamide suspension to rats. The pharmacokinetic parameters revealed enhancement in oral bioavailability when FLT was delivered as niosomal formulation, confirmed with highly significant AUC_0–∞_ with 2 folds increase of FLT-loaded niosomes (741.583 ± 33.557) compared with free FLT suspension (364.536 ± 45.215) that was significant with (*p*-value < 0.001). In addition, FLT maximum plasma concentration (C_max_) of FLT-loaded niosomes (6.950 ± 0.45) was 3 times higher than drug suspension (2.650 ± 0.55) that also highly significant with (*p*-value < 0.001). The previous results may be attributed to niosomal contents delivery to intestinal cells via vesicular endocytosis; also transport of FLT-loaded niosomes through the lymphatic system might be another likely reason to avoid the first hepatic metabolism that greatly affects the free Flutamide [55]. Additionally, the FLT clearance from the niosomal system was significantly 2 times lower than the free suspension with (*p*-value < 0.05) that may be attributed to the reduction of the tissue uptake by evading the reticuloendothelial system.

## 4. Conclusions

Niosomes, as an encouraging drug delivery system, have revealed excellent consequences in the treatment of cancer in the last few years. Based on this revolt in fighting cancer, our research demonstrated the application of niosomal system to improve the oral bioavailability of a sparingly water-soluble drug such as FLT. The Draper–Lin small composite design was applied to optimize the particle size (748.6 nm), ζ potential (−48.60 mV), EE% (72.8%) and cumulative release after 24 h (72.2%) of the FLT-loaded niosomes by selecting Span 60 at 1 M, cholesterol at 0.93 M, DCP at 23 mg and FLT at 6.6 mg. The optimized FLT niosomes show great selectivity toward cancer cells with IC_50_ of 0.64 ± 0.04 µg/mL without affecting the normal body cells with IC_50_ > 100 µg/mL. Additionally, the ex vivo permeability via rabbit intestine and in vivo oral administration to rats have shown improved FLT solubility and absorption after being involved into the niosomal system which indicates higher bioavailability of the FLT-loaded niosomes in comparison to the unprocessed FLT powder. This enhancement in bioavailability can be applied in the future to reduce the administrating dose of FLT and so reduce its related adverse effects. The niosomal approach can also be considered to other BCS class II drugs. One should also consider the intake of total cholesterol per day using a niosomal system.

## Figures and Tables

**Table 1:** Factors and responses of D-LSCD for development of flutamide-loaded niosomes.

Independent Variables (Factors)	Levels			Units
	Low (−1)	Medium (0)	High (+1)	
X_1_: Span 60 concentration	1	2	3	Molar
X_2_: Cholesterol concentration	0.5	1	1.5	Molar
X_3_: DCP concentration	10	15	20	mg
X_4_: Drug concentration	5	7.5	10	mg
Dependent variables (Responses)	Units		Goal	
Y_1_: Mean particle size	nm		Minimize	
Y_2_: Zeta potential	mV		Maximize	
Y_3_: Entrapment efficiency	%		Maximize	
Y_4_: Cumulative release after 24 h	%		Maximize	

**Table 2:** Components of the 18 formulations generated by D-LSCD for preparation of FLT loaded niosomes and their detected responses (Y_1_–Y_4_).

Run	X_1_ (M)	X_2_ (M)	X_3_ (mg)	X_4_ (mg)	Y_1_ (nm)	Y_2_ (mV)	Y_3_ (%)	Y_4_ (%)
1	2.0	0.16	15.0	7.5	425.7	25.74	69.06	94.03
2	2.0	1.0	15.0	7.5	609.9	23.45	77.16	87.39
3	2.0	1.0	15.0	3.3	208.2	32.37	93.34	77.02
4	3.0	1.5	20.0	5.0	179.4	44.44	80.61	68.23
5	2.0	1.0	23.41	7.5	359.9	46.74	79.59	79.50
6	3.68	1.0	15.0	7.5	204.8	25.17	78.62	94.38
7	1.0	0.5	10.0	5.0	657.6	26.06	86.91	87.15
8	2.0	1.84	15.0	7.5	925.4	25.35	79.39	62.75
9	1.0	1.5	10.0	10.0	1068.6	10.46	72.58	73.49
10	0.32	1.0	15.0	7.5	1087.9	18.52	73.58	90.19
11	3.0	0.5	20.0	10.0	1087.9	28.85	73.23	92.42
12	2.0	1.0	15.0	11.7	893.0	17.79	68.22	94.23
13	2.0	1.0	15.0	7.5	600.6	23.61	77.76	86.72
14	3.0	0.5	10.0	10.0	1140.8	14.56	48.37	84.29
15	1.0	0.5	20.0	5.0	211.6	46.76	87.82	89.23
16	1.0	1.5	20.0	10.0	1293.4	28.62	70.05	79.28
17	3.0	1.5	10.0	5.0	685.6	25.94	84.09	71.64
18	2.0	1.0	6.59	7.5	1063.9	10.47	68.39	68.89

Note: The observed values of Y_1_–Y_4_ represent the means of three determinations; standard deviations were <5% of the mean and thus are omitted from the table.

**Table 3:** Statistical ANOVA results of the responses (Y_1_–Y_4_).

Responses	Y_1_		Y_2_		Y_3_		Y_4_	
Factors	Effect	*p* Value	Effect	*p* Value	Effect	*p* Value	Effect	*p* Value
X_1_	−525.094	0.0298 *	3.95412	0.1794	2.99986	0.0359 *	2.49156	0.1767
X_2_	297.122	0.1142	−0.23191	0.9249	6.14523	0.0050 *	−18.6016	0.0010 *
X_3_	−287.663	0.0450 *	19.4259	0.0009 *	5.64896	0.0018 *	4.45749	0.0163 *
X_4_	407.183	0.0564	−8.66933	0.0315 *	−14.9383	0.0004 *	10.2284	0.0055 *
X_1_^2^	104.226	0.3453	0.117679	0.9450	−1.15202	0.1378	3.65883	0.0337 *
X_1_X_2_	−306.942	0.1791	6.50817	0.1154	3.86321	0.0373 *	6.9211	0.0333 *
X_1_X_3_	−84.475	0.5094	−1.5175	0.4840	5.75013	0.0037 *	−0.787353	0.5557
X_1_X_4_	264.847	0.2289	1.46058	0.6557	3.39176	0.0515	−3.49076	0.1556
X_2_^2^	124.873	0.2731	2.73396	0.1803	−2.47802	0.0228 *	−6.17171	0.0081 *
X_2_X_3_	54.375	0.6636	0.4175	0.8406	−7.9462	0.0014 *	−1.95471	0.1992
X_2_X_4_	−490.719	0.0683	3.48162	0.3245	10.7626	0.0021 *	5.63016	0.0557
X_3_^2^	150.576	0.2049	4.89768	0.0526	−2.6466	0.0191 *	−9.13476	0.0026 *
X_3_X_4_	281.025	0.0889	−1.6875	0.4411	6.22351	0.0029 *	3.80851	0.0494 *
X_4_^2^	36.5197	0.7216	2.40517	0.2234	2.15683	0.0328 *	−1.05797	0.3602
R^2^	96.8156	-	98.9129	-	99.827	-	99.5018	-
Adj-R^2^	81.955	-	93.8397	-	99.0196	-	97.1767	-
SEE	159.964	-	2.69504	-	0.982157	-	1.68392	-
MAE	55.4977	-	0.931459	-	0.329098	-	0.510918	-

Note: * Significant effect of factors on individual responses at *p*-value < 0.05.Abbreviations: X_1_, span 60 concentration; X_2_, the concentration of cholesterol; X_3_, the DCP concentration; X_4_, the drug concentration; X_1_X_2_, X_1_X_3_, X_1_X_4_,X_2_X_3_, X_2_X_4_ and X_3_X_4_ the interaction term between the factors; X_1_^2^, X_2_^2^, X_3_^2^ and X_4_^2^ the quadratic terms between the factors; R^2^, R-squared; Adj-R^2^, adjusted R-squared; SEE, standard error of estimate and MAE, mean absolute error.

**Table 4:** Correlation coefficients (r) obtained by different kinetic release models for all FLT-loaded niosomal formulations (F1–F18).

Formula	Zero	First	Second	Diffusion	Hixon	Baker	Chosen (r)
F1	0.77884	−0.3130	0.99391	0.91929	0.86902	0.91341	Second
F2	0.79987	−0.15225	0.97267	0.92826	0.86614	0.91872	Second
F3	0.77575	0.02235	0.88952	0.92672	0.83232	0.89964	Diffusion
F4	0.76047	0.09493	0.80290	0.91523	0.79883	0.86138	Diffusion
F5	0.89667	−0.0236	0.97101	0.98107	0.94607	0.98595	Baker
F6	0.80957	−0.3519	0.98709	0.93543	0.88458	0.91220	Second
F7	0.73088	−0.095	0.88026	0.89385	0.78219	0.82424	Diffusion
F8	0.80532	0.10430	0.80277	0.93812	0.83312	0.87960	Diffusion
F9	0.81232	0.00632	0.83747	0.93253	0.83414	0.85195	Diffusion
F10	0.78345	−0.21068	0.96886	0.92156	0.85012	0.89149	Second
F11	0.89872	−0.24446	0.97099	0.97609	0.96952	0.99373	Baker
F12	0.85848	−0.33234	0.99074	0.95456	0.92953	0.95351	Second
F13	0.79982	−0.14013	0.96851	0.92827	0.86438	0.91751	Second
F14	0.89572	−0.05584	0.96065	0.97780	0.95615	0.98393	Baker
F15	0.96825	−0.11995	0.92649	0.98927	0.98630	0.95317	Diffusion
F16	0.91035	−0.03837	0.97613	0.97601	0.95236	0.98378	Baker
F17	0.68996	0.06359	0.71193	0.86348	0.70666	0.72627	Diffusion
F18	0.94105	0.08453	0.92071	0.99799	0.97502	0.99713	Diffusion
OF	0.76139	0.046344	0.83252	0.91157	0.80075	0.85805	Diffusion
Free FLT	0.97730	0.409971	0.96311	0.87054	0.95333	0.90105	Zero

**Table 5:** Optimal calculated independent variables and observed, predicted and residual values for dependent variables.

Independent Variables	Optimum	Dependent Variables	Predicted Values	Observed Values	Residuals	Prediction Error (%)
Span 60 concentration (X_1_)	1.00	Mean particle size (Y_1_)	736.4	748.6	−12.2	1.66
Cholesterol concentration (X_2_)	0.93	Zeta potential (Y_2_)	46.76	48.60	−1.84	3.93
DCP concentration (X_3_)	23.08	Encapsulation efficiency (Y_3_)	74.5	72.8	1.7	2.28
Drug concentration (X_4_)	6.66	Cumulative drug release after 24h (Y_4_)	78.6	72.2	6.4	8.14

**Table 6:** Calculated pharmacokinetic parameters resulted after oral administration of optimized flutamide-loaded niosomes and free drug suspension.

Pharmacokinetic Parameter	Flutamide Loaded Niosomes	Free Flutamide Suspension
t_1/2_ (min)	163.418 ± 28.485	164.224 ± 31.542
C_max_ (μg/mL)	6.950 * ± 0.45	2.650 ± 0.55
AUC_(0–6)_ (μg/mL × min)	600.125 * ± 57.875	280.550 ± 12.298
AUC_(6–∞)_ (μg/mL × min)	141.458 ± 24.318	83.986 ± 32.917
AUC_(0–∞)_ (μg/mL × min)	741.583 * ± 33.557	364.536 ± 45.215
AUMC_(0–∞)_ (μg/mL × min^2^)	157603.145 *± 12909.983	88926.240 ± 30130.261
MRT_(0–∞)_ (min)	213.309 ± 26.844	239.542 ± 55.854
Vz/F (mg/kg)/(μg/mL)	8.320 * ± 1.815	16.833 ± 1.950
Cl/F (mg/kg)/(μg/mL)/min	0.035 * ± 0.002	0.072 ± 0.009

Note: * Significantly different from values of free flutamide suspension at *p*-value < 0.05.
